# Ion channel Piezo1 induces ferroptosis of trabecular meshwork cells: a novel observation in the pathogenesis in primary open angle glaucoma

**DOI:** 10.1152/ajpcell.00173.2024

**Published:** 2024-10-28

**Authors:** Kexin Liu, Jing Xu, Rufei Yang, Feng Wang, Ying Su

**Affiliations:** ^1^Department of Ophthalmology, the Fourth Affiliated Hospital of Harbin Medical University, Harbin, People’s Republic of China; ^2^Eye Hospital, the First Affiliated Hospital of Harbin Medical University, Harbin, People’s Republic of China; ^3^Key Laboratory of Hepatosplenic Surgery, Ministry of Education, the First Affiliated Hospital of Harbin Medical University, Harbin, People’s Republic of China

**Keywords:** ferroptosis, glaucoma, Piezo1, shear stress, trabecular meshwork

## Abstract

This study aims to elucidate the role of Piezo1, a mechanosensitive molecule, in trabecular meshwork cells (TMCs) in the context of primary open angle glaucoma (POAG), a leading cause of irreversible visual impairment. Dysfunction of the trabecular meshwork (TM) is a key factor in the elevated intraocular pressure (IOP) observed in POAG, yet the specific mechanisms leading to TM dysfunction are not fully understood. We performed cell stretching on human trabecular meshwork cells (HTMCs) and pharmacologically activated HTMCs with Yoda1 to study the role of Piezo1 in HTMCs. We focused on assessing cell viability, mitochondrial changes, lipid peroxidation, and the expression of ferroptosis-related targets such as acyl-CoA synthetase long-chain family member 4 (ACSL4) and glutathione peroxidase 4 (GPX4). Cell stretching induces ferroptosis in HTMCs, and this phenomenon is reversed by Piezo1 knockdown. In addition, pharmacological activation of Piezo1 also leads to ferroptosis in HTMCs. Furthermore, inhibiting the JNK/p38 signaling pathway was found to mitigate the ferroptotic response induced by Yoda1, thereby confirming that Piezo1 induces ferroptosis in TMCs through this pathway. Notably, our experiments suggest that Yoda1 may trigger ferroptosis in the TM of mouse eyes. Our findings demonstrate that the Piezo1 pathway is a crucial mediator of ferroptosis in TMCs, providing new insights into the pathogenic mechanisms of glaucoma, particularly POAG. This study highlights the potential of targeting the Piezo1 pathway as a therapeutic approach for mitigating TM dysfunction and managing POAG.

**NEW & NOTEWORTHY** This study is the first to show that cell stretching induces ferroptosis in trabecular meshwork cells (TMCs), dependent on Piezo1 activation. Targeting the Piezo1 pathway offers new therapeutic potential for mitigating trabecular meshwork dysfunction and managing primary open angle glaucoma (POAG). The study also reveals Piezo1 induces ferroptosis via the JNK/p38 signaling pathway.

## Graphical abstract

**Figure:**
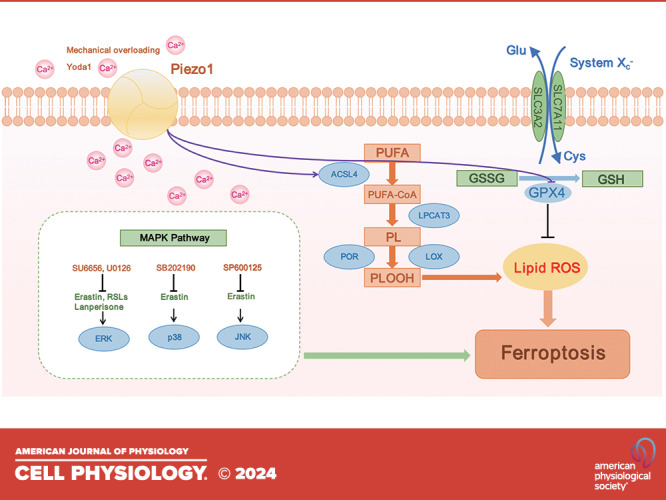

## INTRODUCTION

Glaucoma is a group of blinding eye diseases primarily characterized by pathological elevation of intraocular pressure (IOP) and is the second leading cause of irreversible permanent blindness worldwide (1). The formation of IOP is closely related to the circulation of aqueous humor (AH), and abnormalities in AH dynamics can lead to pathological elevation of IOP, which subsequently damages the optic nerve and retina, resulting in symptoms such as decreased vision and narrowed visual field. In the circulation of AH, the various structural components of the AH outflow pathway are subject to constantly changing hydrodynamics, such as shear stress or tensile forces acting on the walls of the trabecular meshwork (TM) and Schlemm’s canal (SC). Pathological changes in the structure of TM and SC are the primary causes of elevated IOP (1). Mechanotransduction plays a crucial role in the development, physiology, and disease states of the circulatory system. Whether in open-angle or angle-closure glaucoma, if IOP remains persistently elevated, the local homeostasis of TM may be disrupted to varying degrees due to such mechanical stimuli.

Mechanosensitive ion channels are an essential component of the mechanotransduction system. At least 11 types of mechanotransduction channels are expressed in the human TM (2), with Piezo1 being more abundantly detected in human trabecular meshwork cells (HTMCs) (3). In 2010, Coste et al. (4) identified Piezo1 as a mechanically sensitive ion channel protein. Piezo1 can respond to different types of mechanical forces, including pressure, stretch, and shear stress, converting mechanical forces into electrical signals and participating in various biological processes, such as cell differentiation, proliferation, migration, and the function of sensory organs (4–9). Studies have shown that under increased pressure, the shut duration of Piezo1 significantly shortens, whereas the open duration slightly increases. This modulation effectively enhances the channel’s open probability and overall activity. Therefore, an increase in IOP (as a form of mechanical stimulus) is likely to enhance Piezo1 activation. Although further experiments are needed to verify this specifically in trabecular meshwork cells (TMCs), existing mechanistic studies provide strong theoretical support for this hypothesis.

Recent studies suggest that the expression and function of Piezo1 in TMCs may be related to the development of primary open-angle glaucoma (POAG). Piezo1 channel mediates the mechanotransduction function of TMCs and has a regulatory effect on IOP and AH outflow (10, 11). In TMCs, mechanical stretching induces intracellular calcium influx by activating the Piezo1 channel, leading to the release of prostaglandin E2 (PGE2) extracellularly (3). Activation of Piezo1 can also lead to reduced fibronectin and increased prostaglandin F2 alpha (PGF2α) secretion through the arachidonic acid cascade (3, 11, 12). In addition, previous studies have indicated that Piezo1 may influence cell survival and function (11), though its specific impact on TM function has yet to be clarified. TMCs in patients with POAG are insensitive to shear stress, implying that some mechanotransduction pathways may be impaired in the TM of patients with glaucoma (13). In this study, we found a downregulation of *Piezo1* expression in the TM tissues of patients with POAG through the analysis of data from public databases, suggesting that the dysfunction of Piezo1 might be associated with the pathogenesis of POAG. To further investigate the function of Piezo1 in TMCs, this study not only activated Piezo1 using Yoda1 but also applied mechanical stretching to the cells, and used either Piezo1 inhibitors or Piezo1 knockdown (Piezo1_KD) to explore the potential role of mechanical stress and Piezo1 in cell survival and function. The results of this study will help elucidate the role of Piezo1 in the pathogenesis of POAG and provide new perspectives and potential targets for the prevention and treatment of glaucoma.

## METHODS AND MATERIALS

### Antibodies and Reagents

Rabbit Piezo1 antibody (15939-1-AP; Proteintech), rabbit acyl-CoA synthetase long-chain family member 4 (ACSL4) antibody (22401-1-AP; Proteintech), mouse glutathione peroxidase 4 (GPX4) antibody (67763-1-Ig; Proteintech), rabbit SAPK/JNK antibody (No. 9252; Cell Signaling Technology), rabbit phospho-SAPK/JNK (Thr183/Tyr185) (81E11) (No. 4668; Cell Signaling Technology), p38 MAPK antibody (No. 9212; Cell Signaling Technology), phospho-p38 MAPK (Thr180/Tyr182) antibody (No. 9211; Cell Signaling Technology), rabbit β-actin antibody (20536-1-AP; Proteintech) were used. Yoda1 (HY-18723; MCE), Ferrostatin-1 (Fer-1) (S7243; Selleck), GsMTx4 (HY-P1410; MCE) and SP600125 (HY-12041; MCE) were used. All antibodies used in this study have been validated through knockdown/knockout experiments (14–20).

### Cell Culture

Primary human trabecular meshwork cells (HTMCs) were obtained from ScienCell Research Laboratories (ScienCell, Carlsbad, CA). HTMCs are cultured using a specialized trabecular meshwork cell medium (TMCM; ScienCell, Carlsbad, CA). Culturing conditions are maintained at 37°C with 5% CO_2_. Experiments are conducted using HTMCs between the 5th and 8th passage to avoid cellular senescence. Cells were treated with varying concentrations of drugs determined by experimental groups, ensuring the dimethyl sulfoxide (DMSO) content is consistent across all groups and remains below 0.1%.

### Generation of Stable Cells Using Lentiviral Infection

To establish a stable knockdown of the *Piezo1* gene in HTMCs, a lentiviral vector (GV493) containing short hairpin RNA (shRNA) targeting *Piezo1*, purchased from Genechem (Shanghai, PR China), with the following elements: hU6-MCS-CBh-gcGFP-IRES-puromycin, was used. The target sequence was 5′-GCGTCATCATCGTGTGTAAGA-3′, which specifically knocked down the expression of the *Piezo1* gene. HTMCs were seeded into 6-well plates at a density of 1 × 10^5^ cells per well and allowed to adhere overnight. The lentivirus containing the *Piezo1*-targeting sequence was added at a multiplicity of infection (MOI) of 10, and cells were incubated for 12–16 h at 37°C in a 5% CO_2_ atmosphere. After infection, the medium was replaced with fresh complete medium, and cells were cultured for an additional 48 h. Puromycin (2 µg/mL; A1113802, Gibco) was then added to the culture medium to select infected cells. The medium containing puromycin was refreshed every 48 h for a total of 5 days, ensuring that noninfected cells were eliminated. Knockdown efficiency of Piezo1 was confirmed by Western blotting. For the control, lentivirus containing a noneffective shRNA sequence (CON313, 5′- TTCTCCGAACGTGTCACGT-3′) was used under the same conditions to account for nonspecific effects of the lentivirus or puromycin.

### Cell Characterization

A phase-contrast micrograph of HTMCs at *passage 5* was captured in a state of stable confluence. The inducibility of myocilin expression in HTMC by dexamethasone (DEX) was confirmed in line with prior studies (21). In brief, HTMCs at 90% confluence were incubated with 100 nM DEX in freshly changed medium, with the medium being replaced bi-daily. Following a 7-day treatment period, Western blot was conducted to evaluate the production of myocilin.

### Cell Stretching

HTMCs were seeded onto stretch chambers (2 cm × 2 cm; Menicon Life Science, Nagoya, Japan) with high cell adhesion properties and cultured in trabecular meshwork cell medium (TMCM; ScienCell, Carlsbad, CA). After the HTMCs had grown and completely covered the bottom, the chamber was stretched cyclically using a ShellPa Pro Cell Stretching System (Menicon Life Science, Nagoya, Japan) and the adherent HTMCs on the chambers underwent a fixed frequency of stretching. The stretching period was set to 8 s/cycle (0.125 Hz), the amplitude was 20% of the original length. After stretching, the HTMCs media was collected, cleared by centrifugation (400 *g*, 3 min) and kept at −80°C as conditioned media for further experiments.

### Intracellular Ca^2+^ Measurements

Intracellular calcium was measured using Fluo-4 AM (S1060, Beyotime, PR China), diluted to 5 μM with Hank’s balanced salt solution (HBSS). After incubating the cells with the dye at 37°C for 30 min and washing them, added the corresponding drug or applied mechanical stretching. The fluorescence microscope facilitated the observation of intracellular Ca^2+^ influxes. A confocal microscope was used to monitor real-time Ca^2+^ influxes.

### Immunofluorescence

HTMCs were fixed with 4% paraformaldehyde for 15 min, permeabilized with 0.5% Triton X-100 for 10 min, blocked with 5% bovine serum albumin (BSA), incubated with antibodies for 2 h, washed with phosphate-buffered saline (PBS) three times, and then incubated with Alexa Fluor 488 (or 594)-conjugated secondary antibodies (Invitrogen) for 1 h. Subsequently, the cells were stained with host pen cyclic peptide (abs47048273, Absin) for 1 h. Finally, the cells were counterstained with 4',6-diamidino-2-phenylindole (DAPI) and detected using fluorescence microscopy (×60, EVOS M5000).

### Cell Viability Assay

Cells were seeded into a 96-well plate or stretching chambers and precultured in an incubator (37°C, 5% CO_2_) to allow for attachment. After finishing the treatment on the cells, fresh medium containing 10% Cell Counting Kit-8 (CCK-8) reagent (Seven, SC119) was added. Absorbance at 450 nm is measured using a microplate reader after an incubation period of 4 h.

### Cell Proliferation Assay

Cell proliferation experiments were performed using a 5-ethynyl-2′-deoxyuridine (EdU) assay kit (C10310-1, RiboBio). In summary, the cells were treated with 10 mM EdU for 2 h at 37°C, fixed with 4% paraformaldehyde for 30 min, incubated with 2 mg/mL glycine for 5 min, washed with PBS, and treated with 0.5% Triton X-100 for 10 min. Then, 100 μL of 1× Apollo was added, and the cells were washed with PBS again. Next, the cells were observed in a fluorescence microscopy (×10, Nikon, Japan).

### Western Blot Analysis

Proteins were extracted from cells and immunoprecipitated using radio immunoprecipitation assay (RIPA) lysis buffer (Beyotime, PR China). Total protein (20–40 μg) was subjected to 7.5%, 10%, and 12.5% SDS–PAGE and transferred to nitrocellulose membranes. The membranes were blocked with 5% skim milk and incubated with antibodies overnight at 4°C. The second day, the membranes were washed and incubated with secondary antibodies (Santa Cruz Biotech). The bands were visualized with the Molecular Imager System (Bio-Rad). Primary antibodies used were Piezo1 (dilution 1:500), ACSL4 (dilution 1:2,000), GPX4 (dilution 1:1,000), SAPK/JNK (dilution 1:1,000), p-SAPK/JNK (dilution 1:1,000), p38 MAPK (dilution 1:1,000), p-p38 MAPK (dilution 1:1,000) and β-actin (dilution 1:5,000).

### Transmission Electron Microscopy

HTMCs were fixed in 2.5% glutaraldehyde, washed three times with 0.1 M orthophosphoric acid, postfixed in 1% osmium tetroxide buffer, washed three times again with 0.1 M orthophosphoric acid, dehydrated with different concentrations of acetone, embedded in spur resin, and cut into thin sections. The sections were stained with a saturated solution of uranyl acetate and lead citrate. Sections were examined using transmission electron microscopy (TEM) (HITACHI H-7650) at 80 kV.

### Reactive Oxygen Species Detection

The reactive oxygen species (ROS) was detected using a ROS assay kit (S0033S; Beyotime, PR China). The HTMCs were seeded in 24-well plates at 10,000 cells/well and treated with appropriate drugs after cell attachment. Then HTMCs were stained with 5 μM 2′,7′-dichlorofluorescein diacetate (DCFH-DA) probe for 30 min according to the manufacturer’s instructions. The stretched HTMCs were stained in cell stretching chambers. HTMCs were visualized using a fluorescence microscopy (×10, Nikon, Japan).

### Malondialdehyde Assay

According to the manufacturer’s instructions, the relative malondialdehyde (MDA) concentration in HTMCs was assessed by Lipid Peroxidation Assay Kit (S0131S, Beyotime). Briefly, the HTMCs were seeded in culture dishes (100 mm diameter) at 1 × 10^6^ cells/well and treated with appropriate drugs. The stretched HTMCs were seeded in cell stretching chambers. Then the HTMCs were treated with MDA lysis buffer and reacted with thiobarbituric acid (TBA) to generate an MDA-TBA adduct according to the manufacturer’s instructions. The absorbance of the MDA-TBA adduct at 532 nm was detected.

### Lipid ROS Detection

The lipid ROS was detected using a BODIPY 581/591 C11 kit (Thermo Fisher Scientific, Waltham, MA). The HTMCs were seeded in 24-well plates at 10,000 cells/well for 24 h. Then HTMCs were stained with 10 μM C11-BODIPY (581/591) probe for 30 min according to the manufacturer’s instructions. HTMCs were visualized using fluorescence microscopy (×10, Nikon, Japan). The oxidized BODIPY (O-BODIPY) were observed at excitation/emission wavelengths of 488/510 (traditional FITC filter set), whereas the reduced BODIPY (R-BODIPY) were observed at excitation/emission wavelengths of 581/591 nm (Texas Red filter set).

### Intraocular Injection of Drugs in C57BL/6J Mice

The experimental protocol was approved by the Institutional Review Board of the First Affiliated Hospital of Harbin Medical University (IACUC No. 2023062). Male C57BL/6J mice (8 wk old) were randomly divided into three groups, with eight mice in each group. The mice in the vehicle group were injected with 2 μL of PBS solution containing DMSO in the anterior chamber; mice in the Yoda1 group were injected with 2 μL of Yoda1 (40 µM) in the anterior chamber; mice in the Fer-1 group were injected with 2 μL of Yoda1 (40 µM) and Fer-1 (40 µM) in the anterior chamber. The volume of DMSO used in each group was equal (0.18%). The observation period was 7 days, during which the anterior chamber was injected every morning at 9:00 AM. After anesthesia with 150–200 µL of 1.25% Avertin via intraperitoneal injection, the periocular area was disinfected with 1% povidone-iodine, followed by conjunctival sac rinsing. Anterior chamber injections were performed at the limbus of the transparent cornea using a sterile microsyringe (30 G 1/2-in. sterile needle, Hamilton, 10 µL), avoiding the lens and iris. Postoperative antibiotic ointment was applied. Mice were then allowed to recover before being transferred back to their housing cages for routine rearing.

### Tissue Immunofluorescence

Animals were perfused with 4% paraformaldehyde for fixation, followed by enucleation of the eyeballs half an hour later and further fixation in 4% paraformaldehyde for 48 h. After gradient dehydration (10% sucrose for 1 h, 20% sucrose for 1 h, 30% sucrose overnight), samples were embedded in optimal cutting temperature compound (OCT) and cryosectioned. After cryosectioning, the samples were fixed in −20°C cold methanol for 15 min, transferred to room temperature PBS for staining, and washed three times with PBS. Then, permeabilized with 0.3% Triton-X 100 for 15 min, followed by two PBS washes. Blocked with 10% goat serum at room temperature for 60 min. After removing the blocking solution, sections were incubated with primary antibodies diluted in 5% goat serum at 4°C overnight. Washed four times with 1% goat serum, followed by incubation with secondary antibodies diluted in 5% goat serum in a dark environment for 2 h, and then washed four times in 1% goat serum in the dark. After removing the secondary antibodies, DAPI was applied, covered with a coverslip, and mounted. A confocal microscope was used for observation and image collection.

### RNA Extraction and RNA-Sequencing

Total RNA was extracted using the TRIzol method (Invitrogen, CA) and treated with RNase-free DNase I (Takara, Kusatsu, Japan). RNA degradation and contamination were monitored on 1% agarose gels. RNA was quantified using Agilent 2100 Bioanalyzer (Agilent Technologies, CA) and the quality and integrity were assessed by NanoDrop spectrophotometer (Thermo Scientific, DE). A total amount of 1.5 μg RNA per sample was used as input material for the RNA sample preparations. Sequencing libraries were generated using NEBNext UltraTM RNA Library Prep Kit for Illumina (NEB) following manufacturer’s recommendations and index codes were added to attribute sequences to each sample. The library preparations were sequenced on an Illumina Novaseq 6000 platform by the Beijing Allwegene Technology Company Limited (Beijing, People’s Republic of China) and paired-end 150 bp reads were generated.

### Statistical Analysis

Statistical and image analyses were conducted using Prism 9.0 (GraphPad) and ImageJ software. Data are presented as the means ± SD of three replicates. Statistical comparisons were performed using SPSS statistical software (IBM, Armonk, NY), with the Student’s *t* test and Dunnett’s test for comparisons among the groups. A *P* value <0.05 was considered statistically significant.

## RESULTS

### Reduced mRNA Expression of Piezo1 Observed in the TM of Patients with POAG

Gene expression data and clinical information for TM tissues from patients with POAG and from normal controls are available in the online database GEO, data set GSE27276, which includes transcriptome sequencing results for 19 POAG TM and 17 control TM. A total of 813 mechanosensitive ion channel genes were retrieved from the GeneCards database. These genes were normalized and differentially analyzed using the “limma” package in R software, with a *P* value <0.05 considered to indicate statistically significant differential expression.

**Figure 1.**
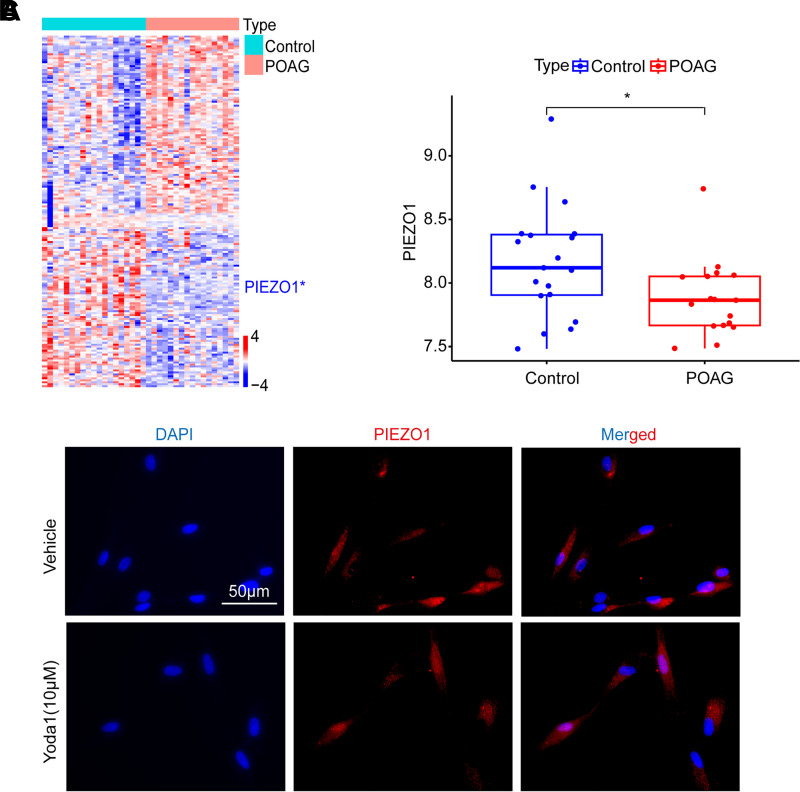
Expression of mechanosensitive ion channel Piezo1 in trabecular meshwork (TM). *A*: differential expression of mechanosensitive ion channel genes between normal human TM tissue (*n* = 17) and TM tissue from patients with primary open angle glaucoma (POAG) (*n* = 19). *B*: reduced expression of *Piezo1* in the TM of patients with POAG. *C*: expression of Piezo1 in human trabecular meshwork cell (HTMCs). Blue represents the nucleus and red represents Piezo1. The DMSO content in each group is consistent and less than 0.1%.

Differential analysis of mechanosensitive ion channel genes in the GSE27276 data set revealed significant changes in the expression of 179 genes within the TM of patients with POAG (Fig. 1*A*), including a notable downregulation of *Piezo1* expression in POAG TM tissues (Fig. 1*B*). This suggests that the TM tissues in patients with POAG are unable to normally detect mechanical stimuli via Piezo1. Previous research has demonstrated that TMCs from human patients with POAG are insensitive to shear stress, suggesting that some mechanotransduction pathways might be impaired in the TM of patients with glaucoma (13). Therefore, the insensitivity of TM tissues to shear stress in patients with POAG may be related to the reduced expression of Piezo1 in the TM tissues of these patients.

### Piezo1 Involvement in Ferroptosis-Related Responses under Cell Stretching in TMCs

**Figure 2.**
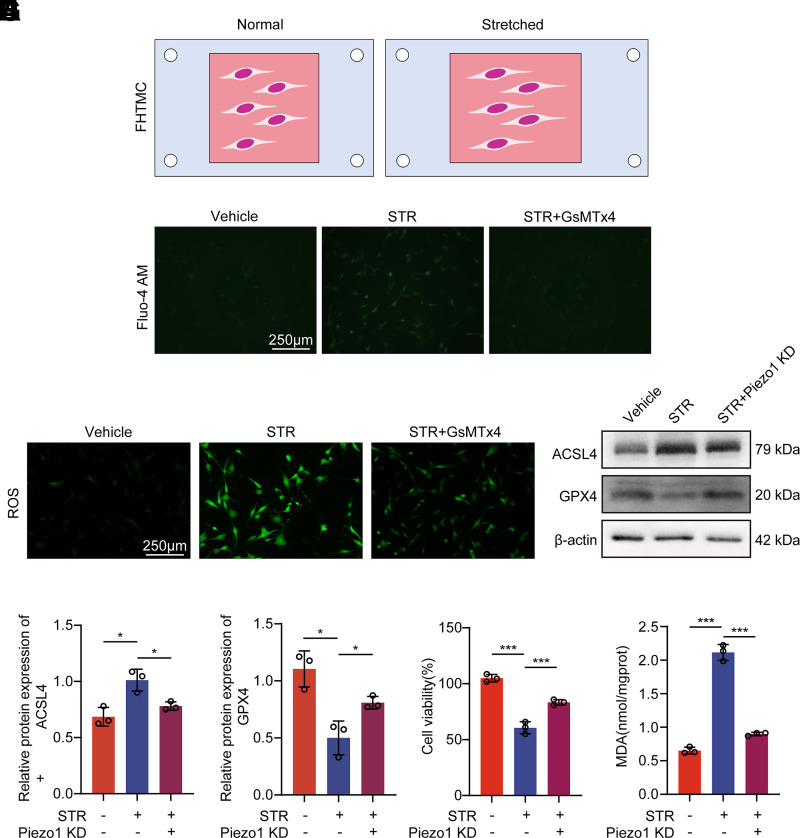
Piezo1 is involved in cell stretch-induced ferroptosis in human trabecular meshwork cells (HTMCs). *A*: mechanical stimulation of HTMCs was performed using a cell stretching chamber. *B*: calcium influx in HTMCs after cell stretching (20%, 10 min). *C*: intracellular reactive oxygen species (ROS) levels in HTMCs after cell stretching (20%, 12 h). *D*–*F*: expression of acyl-CoA synthetase long-chain family member 4 (ACSL4) and glutathione peroxidase 4 (GPX4) protein in HTMCs after cell stretching (20%, 12 h). Data are the means ± SE. *n* = 3 independent repeats. *P < 0.05 (Student’s *t* test). *G*: cell viability of HTMCs after cell stretching (20%, 12 h) was measured using Cell Counting Kit-8 (CCK8) assay in each concentration group. Data are the means ± SE. *n* = 3 independent repeats. ***P < 0.001 (Student’s *t* test). *H*: malondialdehyde (MDA) content of HTMCs after cell stretching (20%, 12 h). Data are the means ± SE. *n* = 3 independent repeats. ***P < 0.001 (Student’s *t* test). The volume of DMSO used in each group is equal and less than 0.1%.

TMCs are frequently subjected to mechanical forces and deformation caused by changes in IOP and eye movements. So far, there has been no definitive research indicating whether mechanical stimuli such as cell stretching can induce ferroptosis in TMCs, either under physiological or pathological conditions, or how this process might occur. In our study, we used a cell stretcher to apply different stretching conditions (Fig. 2*A*) and investigated whether cell stretching could induce ferroptosis. A 20% stretch was applied to the HTMCs, resulting in increased Ca^2+^ influx (Fig. 2*B*). After 12 h of 20% stretching, the ROS (Fig. 2*C*) and lipid peroxidation levels (Fig. 2*H*) in the stretched cells increased. Ferroptosis-related proteins showed a ferroptotic trend, with increased ACSL4 and decreased GPX4 expression. These effects were reversed by the ion channel inhibitor GsMTx4 (5 μM) or Piezo1_KD (Fig. 2, *D–F*, Supplemental Fig. S1). In addition, reducing extracellular calcium concentration reversed the changes in ACSL4 and GPX4 protein expression induced by cell stretching, suggesting that calcium influx may play a partial role in the ferroptosis process of HTMCs triggered by mechanical stretching (Supplemental Fig. S2). We have demonstrated that TMCs undergo ferroptosis when subjected to a certain degree of mechanical stimulation, and that Piezo1 is involved in TMC ferroptosis induced by cell stretching.

### Yoda1-Induced Activation of Piezo1 Results in Calcium Influx and Lipid Peroxidation in HTMCs

**Figure 3.**
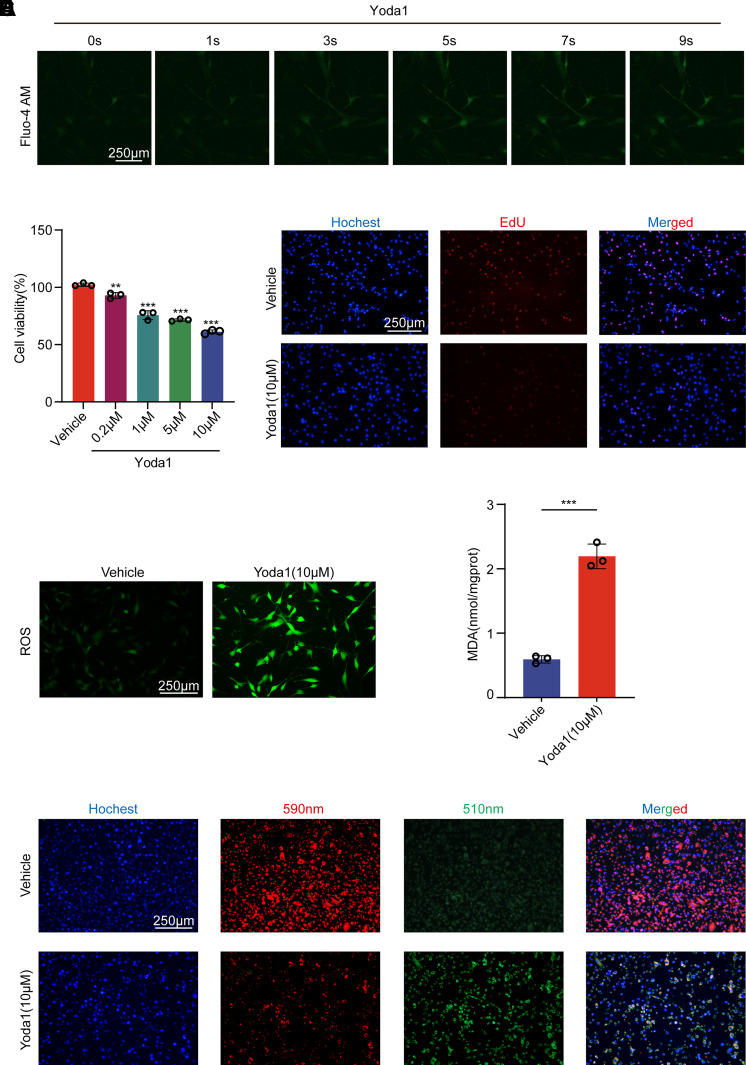
Effects of Yoda1 treatment on calcium influx, cell viability, proliferation, reactive oxygen species (ROS) levels, malondialdehyde (MDA) content, and lipid peroxidation in human trabecular meshwork cells (HTMCs). *A*: dynamic changes in calcium influx at 1, 3, 5, 7, and 9 s after the addition of Yoda1 (10 μM). The intensity of the green fluorescence signal represents the intracellular calcium concentration. *B*: cell viability of HTMCs treated with Yoda1 (0.2 μM, 1 μM, 5 μM, 10 μM) for 24 h was measured using Cell Counting Kit-8 (CCK8) assay in each concentration group. Data are the means ± SE. *n* = 3 independent repeats. **P < 0.01, ***P < 0.001 vs. vehicle group (Dunnett’s test). *C*: 5-ethynyl-2′-deoxyuridine (EdU) assay to test the proliferative capacity of vehicle group and Yoda1 group (10 μM, 24 h). *D*: intracellular ROS levels in vehicle group and Yoda1 group (10 μM, 24 h). *E*: MDA content in vehicle group and Yoda1 group. Data are the means ± SE. *n* = 3 independent repeats. ***P < 0.001 (Student’s *t* test). *F*: the degree of lipid peroxidation in vehicle group and Yoda1group. The volume of DMSO used in each group is equal and less than 0.1%.

Piezo1 can be selectively activated by the small molecule drug Yoda1 in both in vitro and in vivo environments. To investigate the regulatory role of Piezo1 on the function of HTMCs, we conducted further studies using Yoda1. To eliminate the potential effects of the solvent DMSO on the cells, we balanced the concentration of Yoda1 across all groups, ensuring that the content of DMSO was less than 0.1%. Using Fluo-4 AM to detect Ca^2+^ influx, we confirmed that after treatment with the Piezo1 agonist Yoda1, the calcium ion fluorescence intensity in HTMCs was enhanced, indicating an increase in Ca^2+^ influx (Fig. 3*A*). It can be observed that after the application of Yoda1, Ca^2+^ influx increased within a very short period of time. To ascertain the expression of Piezo1 in HTMCs, we performed immunofluorescence staining with an anti-Piezo1 antibody on both vector control groups and HTMCs treated with Yoda1 (10 μM) for 24 h. We observed Piezo1 expression in HTMCs. In addition, although Yoda1 activated the Piezo1 ion channel, it did not increase the protein expression of Piezo1 (Fig. 1*C*). CCK8 assays results indicated that Yoda1 reduced HTMC viability in a concentration-dependent manner (Fig. 3*B*, Supplemental Fig. S3). EdU assay results showed that after 24 h of treatment with Yoda1 (10 μM), there was a significant inhibition of the percentage of EdU-positive cells (Fig. 3*C*). These findings suggest that upon activation, Piezo1 inhibits cell viability and proliferative capacity.

Malondialdehyde (MDA) is a primary product of polyunsaturated fatty acid peroxidation reactions. Furthermore, the BODIPY-C11 probe can specifically detect ferroptosis by measuring the quantity of lipid peroxidation products in the cell membrane. In this study, within HTMCs treated with Yoda1, we observed an increase in ROS levels (Fig. 3*D*), suggesting that activation of Piezo1 might cause oxidative damage to the cells. In addition, in HTMCs treated with Yoda1, an increase in MDA content was also observed (Fig. 3*E*), indicating that lipid peroxidation reactions occurred in the cell membrane following Piezo1 activation, which is evidence of HTMC undergoing ferroptosis. Staining of HTMCs with the C11 BODIPY 581/591 fluorescent probe similarly revealed enhanced lipid peroxidation levels in HTMCs treated with Yoda1 (Fig. 3*F*). These data suggest that the activation of Piezo1 leads to an increase in lipid peroxidation levels in HTMCs, which might trigger ferroptosis.

### Activation of Piezo1 Alters Ferroptosis Marker Proteins in HTMCs, Reversible by Fer-1

**Figure 4.**
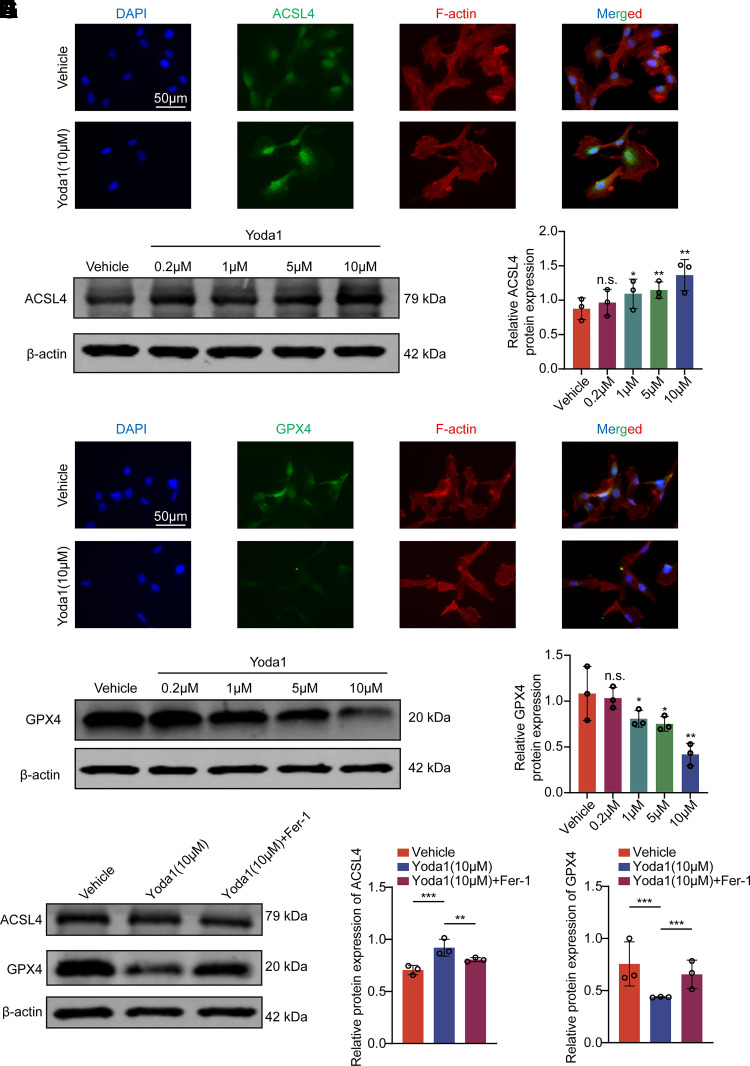
Changes in ferroptosis marker proteins in human trabecular meshwork cell (HTMC) following Piezo1 activation are reversed by ferrostatin-1 (Fer-1). *A*: protein expression of acyl-CoA synthetase long-chain family member 4 (ACSL4) in vehicle group and Yoda1 group. *B* and *C*: expression of ACSL4 protein in vehicle group and Yoda1 group. Data are the means ± SE. *n* = 3 independent repeats. *P < 0.05, **P < 0.01 vs. vehicle group (Dunnett’s test). *D*: protein expression of glutathione peroxidase 4 (GPX4) in vehicle group and Yoda1 group. *E* and *F*: expression of GPX4 protein in vehicle group and Yoda1 group. Data are the means ± SE. *n* = 3 independent repeats. *P < 0.05, **P < 0.01 vs. vehicle group (Dunnett’s test). *G*–*I*: expression of ACSL4 and GPX4 protein in vehicle group, Yoda1 group and Yoda1 + Fer-1 group. HTMCs in Yoda1 + Fer-1 group are treated with both Yoda1 and Fer-1 (10 μM) for 24 h. Data are the means ± SE. *n* = 3 independent repeats. **P < 0.01, ***P < 0.001 (Student’s *t* test). The volume of DMSO used in each group is equal and less than 0.1%.

**Figure 5.**
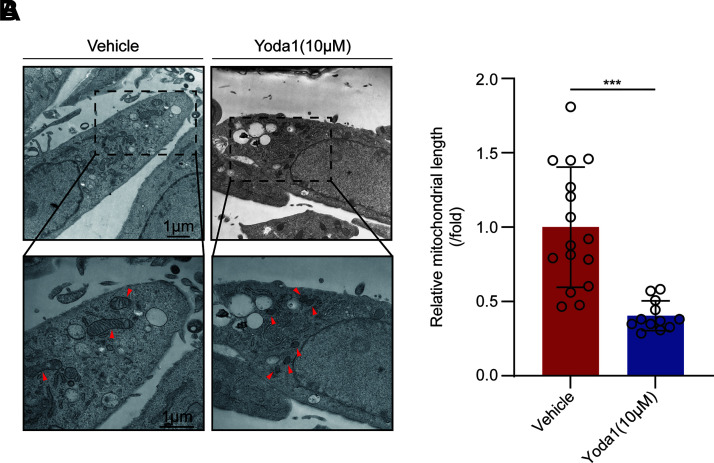
Characteristic changes of ferroptosis occur in human trabecular meshwork cells (HTMCs) following Piezo1 activation. *A* and *B*: observation of vehicle group and Yoda1 group under transmission electron microscopy at various magnifications. Data are the means ± SE. *n* = 3 independent repeats. ***P < 0.001 vs. vehicle group (Student’s *t* test). The volume of DMSO used in each group is equal and less than 0.1%.

Ferroptosis is rigorously regulated by two antagonistic enzymes: ACSL4 and GPX4, which respectively induce and mitigate lipid peroxidation. In this study, varying concentrations of Yoda1 were administered to cells for 24 h to simulate the effects of different shear stress intensities on HTMC, and the expression levels of ACSL4 and GPX4 in HTMCs were examined. With increasing concentrations of Yoda1, the expression of ACSL4 protein in HTMCs was enhanced (Fig. 4, *B* and *C*), and immunofluorescence assays also confirmed that Piezo1 channel activation led to an upregulation of ACSL4 protein expression (Fig. 4*A*). A similar alteration was observed in another ferroptosis marker protein, GPX4, post Piezo1 activation. As the concentration of Yoda1 increased, the expression of GPX4 protein in HTMCs decreased (Fig. 4, *E* and *F*), and immunofluorescence assays corroborated that activation of the Piezo1 channel resulted in a reduction of GPX4 protein expression (Fig. 4*D*). Moreover, the changes in these two ferroptosis marker proteins induced by Piezo1 activation were reversible by the ferroptosis inhibitor Fer-1 (Fig. 4, *G–I*), further substantiating the occurrence of ferroptosis in HTMCs following Piezo1 activation. Previous studies have demonstrated that typical electron microscopic characteristics of cells undergoing ferroptosis include mitochondrial shrinkage and a reduction in mitochondrial cristae. In this study, through TEM observation of HTMCs, we found that compared with control group, the mitochondria in HTMCs treated with Yoda1 exhibited significant shrinkage, a marked decrease in length, and blurred mitochondrial cristae (Fig. 5, *A* and *B*). This also morphologically supports the induction of ferroptosis in HTMCs by Piezo1 activation.

### Ferroptosis-Related Changes in TM Tissues following Piezo1 Activation in Mouse Eyes

**Figure 6.**
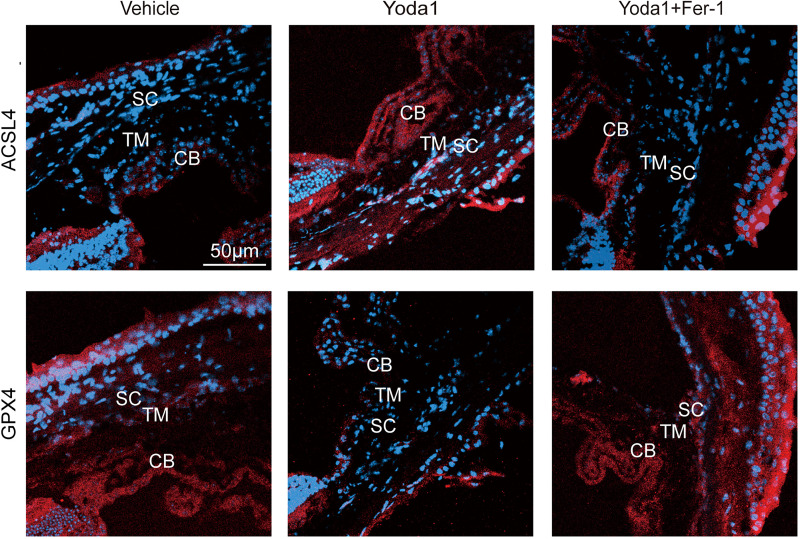
Piezo1 activation induces ferroptosis on trabecular meshwork (TM) tissues in mouse eyes. The mice in the vehicle group were injected with 2 μL of PBS solution containing DMSO in the anterior chamber; mice in the Yoda1 group were injected with 2 μL of Yoda1 (40 µM) in the anterior chamber, and mice in the ferroptosis inhibitor group were injected with 2 μL of Yoda1 (40 µM) and ferrostatin-1 (Fer-1) (40 µM) in the anterior chamber. The volume of DMSO used in each group is equal. The observation period was 7 days, in which the anterior chamber was injected once a day at 9:00 AM. Immunofluorescence staining for acyl-CoA synthetase long-chain family member 4 (ACSL4) and glutathione peroxidase 4 (GPX4) in the eyes of the three groups. CB, ciliary body; SC, Schlemm’s canal.

Ferroptosis occurs following Piezo1 activation, as evidenced by experiments in vivo. Anterior chamber injections were administered to the right eyes of mice every morning at 9:00 AM over a 7-day observation period. The vehicle group received 2 µL of PBS with DMSO, the Yoda1 group received 2 µL of Yoda1 (40 µM), and the ferroptosis inhibitor group received 2 µL of Yoda1 (40 µM) plus Fer-1 (40 µM). On *day 8*, mice were euthanized for cryo-immunofluorescent staining. Immunofluorescent results indicated an upregulation of ACSL4 protein expression and a downregulation of GPX4 protein expression in the TM tissues after Yoda1 injection, which was reversed by Fer-1. This indicates that Yoda1 similarly induces ferroptosis in TM tissues in vivo (Fig. 6).

### JNK/p38 Pathway Involvement in Ferroptosis-like Changes Induced by Piezo1 Activation in HTMCs

**Figure 7.**
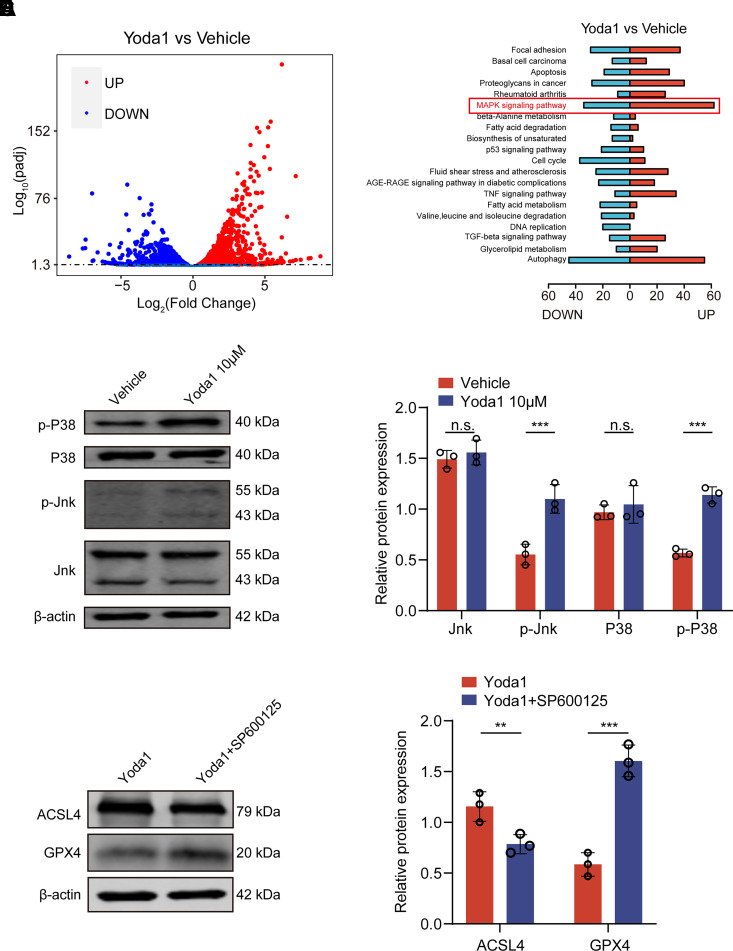
Piezo1 activation induces ferroptosis in human trabecular meshwork cells (HTMCs) via the JNK/p38 pathway. *A*: differentially expressed genes analyzed from transcriptomic sequencing results between vehicle group and Yoda1 group; *n* = 3 independent repeats. *B*: signal pathways identified from KEGG enrichment analysis of the differentially expressed genes. *n* = 3 independent repeats. *C* and *D*: levels of total JNK, total p38, phosphorylated JNK (p-JNK), and phosphorylated p38 (p-p38) protein expression in vehicle group and Yoda1 group. Data are the means ± SE. *n* = 3 independent repeats. ***P < 0.001 vs. vehicle group (Student’s *t* test). *E* and *F*: protein expression levels of acyl-CoA synthetase long-chain family member 4 (ACSL4) and glutathione peroxidase 4 (GPX4) in HTMCs treated with Yoda1 alone and in combination with JNK inhibitor SP600125 for 24 h. Data are the means ± SE. *n* = 3 independent repeats. **P < 0.01, ***P < 0.001 vs. vehicle group (Student’s *t* test). The volume of DMSO used in each group is equal.

To further elucidate the potential role of Piezo1 in HTMCs, we performed transcriptomic sequencing analysis on HTMCs treated with Yoda1. The results indicated that in the HTMCs post-Yoda1 treatment, a total of 4,844 genes exhibited differential expression, comprising 2,332 upregulated genes and 2,152 downregulated genes (Fig. 7*A*). To clarify the specific mechanisms of action of Piezo1 in HTMCs, this study conducted Kyoto Encyclopedia of Genes and Genomes (KEGG) enrichment analysis on these 4,844 differentially expressed genes. The results demonstrated significant activation of pathways related to apoptosis, fatty acid metabolism, autophagy, and MAPK among others following Yoda1 treatment. Within these pathways, the MAPK signaling pathway was most significantly enriched (Fig. 7*B*). Given that numerous studies have established a close relationship between MAPK signaling and ferroptosis (22), we further analyzed whether Piezo1 activation could induce ferroptosis through the MAPK pathway. In this experiment, we assessed the proteins JNK and p38 within the MAPK pathway. Postactivation of HTMCs by Yoda1, a significant increase in the phosphorylation levels of JNK and p38 was observed (Fig. 7, *C* and *D*). Moreover, upon treatment with a JNK inhibitor (SP600125), the increased expression of ACSL4 induced by Yoda1 was decreased, whereas the decreased expression of GPX4 was elevated (Fig. 7, *E* and *F*). This suggests that the activation of ferroptosis in HTMCs by Piezo1 occurs through the activation of the JNK/p38 pathway.

## DISCUSSION

The results of this study provide novel insights into the role of the mechanosensitive ion channel Piezo1 in mechanotransduction and ferroptosis in TMCs. First, a downregulation of *Piezo1* expression was observed in the TM tissues of patients with POAG, suggesting a potential link between Piezo1 dysregulation and impaired mechanotransduction in the pathogenesis of glaucoma. POAG is characterized by elevated IOP and impaired AH outflow, and the mechanotransduction pathways in TMCs are crucial for sensing and responding to mechanical stimuli, potentially playing a key role in maintaining normal IOP. Transcriptomic analysis of TM tissues from patients with POAG (GSE27276 data set) revealed a downregulation of *Piezo1* expression, indicating that Piezo1 might be a critical factor in TM cell dysfunction, potentially leading to the inability of these cells to respond appropriately to mechanical stimuli. Previous studies have shown that TMCs from patients with POAG exhibit reduced sensitivity to mechanical stimuli, such as shear stress, which further supports this hypothesis (13).

Cell stretching is a mechanical stimulus used to simulate changes in IOP. Prior to this study, there was no evidence that mechanical stress could induce ferroptosis in the TM. Our experimental results not only demonstrated that a certain degree of mechanical stress could induce ferroptosis in TMCs but also further revealed the role of Piezo1 in stretch-induced ferroptosis in TMCs. Previous studies applied cyclic mechanical stretch with a strain amplitude of 20% and a frequency of 1 Hz to cultured primary HTMCs, simulating a model of acute sustained IOP elevation (23–25). To simulate the mechanical stress experienced by TM tissues under pathological high IOP, we applied 20% strain with low-frequency periodic stretching (0.125 Hz) for 12 h to cultured HTMCs in vitro in this study. The experiments showed that mechanical stretching induced ferroptosis in HTMCs, characterized by elevated ROS, increased lipid peroxidation, and changes in ferroptosis markers ACSL4 and GPX4. Piezo1 played a critical upstream role in this process, and Piezo1 knockdown or the use of the ion channel inhibitor GsMTx4 reversed these effects, indicating that Piezo1-mediated mechanotransduction is a key event in mechanically induced ferroptosis.

In addition, by using the agonist Yoda1 to activate Piezo1, we further confirmed its role in calcium influx, lipid peroxidation, and ferroptosis. After Yoda1 treatment, we observed a rapid increase in Ca^2+^ influx, decreased cell viability and proliferation, and elevated ROS and lipid peroxidation levels, suggesting that Piezo1 activation may cause oxidative damage in HTMCs. The upregulation of ACSL4 and downregulation of GPX4 further confirmed that Piezo1 induces ferroptosis in HTMCs. The use of the ferroptosis inhibitor Fer-1 reversed the changes in ferroptosis markers caused by Piezo1 activation. In addition, the cells treated with Yoda1 exhibited typical ferroptotic morphological features, such as mitochondrial shrinkage. These results further emphasize the role of Piezo1 in regulating ferroptosis in HTMCs through oxidative stress and lipid peroxidation. Notably, although Yoda1 strongly activated Piezo1, no significant increase in Piezo1 protein expression was observed. However, previous studies have shown that a certain degree of mechanical stimulation can upregulate Piezo1 expression (26), suggesting that mechanical stimulation may act through both the regulation of Piezo1 expression and functional state.

Through in vivo experiments, we further confirmed that Piezo1 activation induces ferroptosis in TM tissues. After anterior chamber injections of Yoda1 into the mouse eye, ferroptosis markers, including increased ACSL4 expression and decreased GPX4 expression, were consistent with the in vitro results. These changes could be reversed by the ferroptosis inhibitor Fer-1, further supporting that Piezo1 activation induces ferroptosis in TM tissues in vivo.

Finally, we also revealed the molecular mechanism by which Piezo1 mediates ferroptosis in HTMCs via the JNK/p38 pathway. Transcriptomic analysis showed significant enrichment of the MAPK signaling pathway after Piezo1 activation, suggesting that this pathway might be involved in Piezo1-mediated downstream responses. Given the known relationship between the MAPK signaling pathway and ferroptosis, we further selected the JNK/p38 pathway for in-depth analysis. Experimental results showed that the phosphorylation levels of JNK and p38 increased significantly after Piezo1 activation, indicating that this pathway may play a role in Piezo1-mediated ferroptosis in HTMCs. To validate this hypothesis, we used the JNK inhibitor SP600125, which reversed the Yoda1-induced upregulation of ACSL4 and downregulation of GPX4. This suggests that Piezo1 may trigger ferroptosis via the JNK/P38 pathway. This finding provides new insights into the role of Piezo1 and the MAPK pathway in ferroptosis in TMCs. Modulating this signaling pathway may serve as a potential therapeutic target, offering new possibilities for interventions in glaucoma-related damage.

Cells in TM tissues are often exposed to mechanical forces and deformations caused by changes in IOP and eye movements. Studies have shown that normal IOP is not constant, but undergoes significant fluctuations during daily ocular activities (27, 28). These fluctuations include transient pressure changes due to blinking and eye movements, which can reach amplitudes of up to 10 mmHg, and smaller, sustained periodic fluctuations associated with ocular pulsations (2–3 mmHg) (28). Under pathological conditions, particularly in patients with POAG, IOP fluctuations may become more pronounced and persist for longer durations. Elevated IOP increases the mechanical stress on the TM, leading to expansion and compression of its three-dimensional elastic network structure (1, 29–31). When pressure increases from 8 mmHg to 30 mmHg, the stretch experienced by TM cells can reach up to 50% (1, 29, 30). Mechanosensitive ion channels, such as Piezo1, are known for their rapid response to mechanical stimuli, and are particularly important given the dynamic mechanical environment of the eye (32, 33). These ion channels play a functional role in the AH outflow pathway (2, 34–36), and Piezo1 is considered an active and sensitive mechanosensor in the TM (26).

TMCs must have adaptive mechanisms to cope with the mechanical stress induced by IOP fluctuations, thereby preventing further damage. Previous studies have shown that when TMCs are exposed to static biaxial strain and elevated pressure, autophagy is activated, suggesting that autophagy might be part of an integrated homeostatic response in TMCs under strain (24). Although ferroptosis is often viewed as a destructive process in pathological mechanisms such as neurodegenerative diseases, acute organ injury, and tumors (37), its protective function in clearing aging or dysfunctional cells and maintaining tissue homeostasis is gaining attention (38, 39). Significant progress has been made in understanding the role of ferroptosis in ocular diseases such as glaucoma, cataracts, and diabetic retinopathy (40–43), and studies suggest that iron intake may increase the risk of glaucoma (44). However, the role of ferroptosis in the AH outflow pathway remains underexplored, particularly regarding the pathological remodeling of TMCs. We hypothesize that ferroptosis may play a key role in the functional regulation and pathological changes of the TM.

Considering the prolonged exposure of the TM to mechanical stress from IOP fluctuations, along with the downregulation of *Piezo1* expression observed in TM tissues of patients with POAG, we propose that Piezo1 may have a dual role in POAG. On the one hand, Piezo1 may be moderately activated under normal conditions, contributing to the mechanical sensing of TMCs and helping to maintain IOP homeostasis. On the other hand, under pathological high IOP conditions, excessive activation of Piezo1 could trigger ferroptosis, aiding in the clearance of damaged or dysfunctional TMCs. However, the downregulation of *Piezo1* expression in the TM of patients with POAG may indicate impaired mechanosensory function in these cells, reducing their ability to respond to mechanical stress, and further compromising IOP regulation. In addition, the decreased *Piezo1* expression could represent an adaptive response to prevent excessive ferroptosis. However, this reduction may also hinder the ability of TMCs to clear dysfunctional cells, exacerbating the progression of glaucoma. Whether this mechanism is protective or detrimental in the context of glaucoma remains to be elucidated through further studies.

In conclusion, our study demonstrates that mechanical stress can induce ferroptosis in TMCs via the Piezo1 channel, and that Piezo1 promotes this process by activating the MAPK signaling pathway. This finding is significant for understanding the relationship between mechanical stress, signal transduction, and cell death mechanisms, and has potential applications in glaucoma research. Future studies can further explore the complex relationship between Piezo1 and ferroptosis in cellular activities, particularly its potential impact on IOP regulation. This could not only help to elucidate the pathogenesis of glaucoma but also provide new strategies for its treatment.

## DATA AVAILABILITY

The data set used and/or analyzed during the current study is available from the corresponding author upon reasonable request.

## SUPPLEMENTAL MATERIAL

10.6084/m9.figshare.27879327 Supplemental Figs. S1–S3: https://doi.org/10.6084/m9.figshare.27879327.

## GRANTS

This work was supported by the Natural Science Grant of the Heilongjiang Province of China under Grants H2018035, LH2020H040, the Innovation and Development Foundation of First Affiliated Hospital of Harbin Medical University under Grant 2018L002, and the special grant of Fourth affiliated Hospital of Harbin Medical University Grant HYDSYTB202209.

## DISCLOSURES

No conflicts of interest, financial or otherwise, are declared by the authors.

## AUTHOR CONTRIBUTIONS

K.L., F.W., and Y.S. conceived and designed research; K.L. and J.X. performed experiments; K.L., J.X., and R.Y. analyzed data; K.L. interpreted results of experiments; K.L., J.X., and R.Y. prepared figures; K.L. and J.X. drafted manuscript; K.L., F.W., and Y.S. edited and revised manuscript; K.L., J.X., R.Y., F.W., and Y.S. approved final version of manuscript.
